# Hereditary cancer testing in a diverse sample across three breast imaging centers

**DOI:** 10.1007/s10549-023-07137-1

**Published:** 2023-10-20

**Authors:** Laura Westbrook, Darlene Miltenburg, Vivienne Souter, Melissa K. Maisenbacher, Katherine L. Howard, Youbao Sha, Maygol Yavari, Nicholas Kypraios, Angel Rodriguez, Jeffrey N. Weitzel

**Affiliations:** 1 Natera, Inc., 13011 McCallen Pass, Austin, TX 78753 USA; 2 The Breast Health Institute of Houston, Houston, TX 77054 USA; 3 Precision Prevention, The Kansas University Comprehensive Cancer Center, Kansas City, KS 66160 USA

**Keywords:** Breast cancer, Hereditary cancer genes, Cancer risk assessment, Genetic testing, Pathogenic variants

## Abstract

**Purpose:**

Up to 10% of all breast cancers (BC) are attributed to inherited pathogenic variants (PV) in BC susceptibility genes; however, most carriers of PVs remain unidentified. Here, we sought to determine the yield of hereditary cancer gene PVs among diverse women attending breast imaging centers, who could benefit from enhanced surveillance and/or risk reduction interventions.

**Methods:**

This cross-sectional retrospective cohort study included consecutive women, unselected for personal or family cancer history, who were offered genetic testing for hereditary cancer genes at the time of breast imaging at three centers (November 2020–March 2022).

**Results:**

Among 1943 patients (median age: 66 years), self-reported race/ethnicity was White (34.5%), Hispanic (27.7%), African American (17.9%), Asian (4.5%), Ashkenazi Jewish (0.6%), Other (3.5%), and missing (13.0%). Thirty-nine patients (2%) were identified as carriers of a PV in an autosomal dominant clinically actionable hereditary breast and ovarian cancer (HBOC)-related or Lynch syndrome gene, most frequently, *BRCA2* (6/39; 15.4%), *PALB2* (8/39; 20.5%), *CHEK2* (10/39; 25.6%), and *PMS2* (5/39; 12.8%). Of the 34 PVs with known race/ethnicity, 47% were detected among non-White patients. Overall, 354/1,943 (18.2%) of patients met NCCN guidelines for HBOC gene testing and only 15/39 (38.5%) patients with an autosomal dominant clinically actionable PV met guidelines.

**Conclusion:**

This population health approach extended the reach of genetic cancer risk assessment in a diverse population and highlighted the limits of a guideline-based approach. This may help address inequity in access to risk-appropriate screening and cancer prevention.

## Introduction

A substantial proportion of cancers are associated with pathogenic variants (PV) in hereditary cancer genes [1, 2], including an estimated 10% of breast, 10% of colon, and 20% of ovarian cancers. Approximately, 1 in 300 to 500 people in the population will carry a PV in either *BRCA1* or *BRCA2* (*BRCA*; the genes most commonly associated with hereditary breast and ovarian cancer (HBOC)) [3–6], and 1 in 370 individuals will carry a pathogenic variant (PV) in one of the Lynch syndrome genes (the genes most commonly associated with hereditary colorectal cancer) [7]. Early identification of a PV in a cancer susceptibility gene provides individuals with the opportunity for enhanced surveillance and risk-reducing interventions which can significantly reduce the morbidity and mortality of these cancers [8–13]. Identification of carrier status also provides the opportunity for cascade testing in relatives [14–16].

Genetic testing for cancer susceptibility genes is currently offered predominantly to individuals who are considered high-risk for either *BRCA* or Lynch syndrome based on clinical and family history criteria. However, only around half of the carriers of PVs in *BRCA* and Lynch syndrome genes will meet clinical and family history criteria for genetic testing [5, 17, 18]. Additionally, current approaches to testing for hereditary cancer genes are associated with inequities in referral and access to testing [19, 20].

An alternative approach is population-based hereditary cancer genetic testing which may provide a more clinically effective strategy for early identification of high-risk individuals [5, 21]. To date, population-based testing has been primarily studied in the context of *BRCA* testing in the Ashkenazi Jewish population [22, 23], and data are limited in the general population, especially among under-represented racial and ethnic groups. The objective of this study was to determine the yield of hereditary cancer gene PVs among unselected diverse women attending breast imaging centers, as a potential strategy for more complete identification of high-risk individuals who could benefit from enhanced surveillance and/or risk reduction interventions.

## Materials and methods

### Study population

This retrospective cohort study included unselected female patients who were offered and underwent genetic testing at the time of breast imaging at three imaging centers (Memorial MRI and Diagnostics, Texas) from November 2020 through March 2022. All patients arriving at the imaging centers were given a written flier with an invitation to undergo genetic testing for a panel of hereditary cancer genes. Patients were also offered the option of an online genetic information session with a board-certified genetic counselor prior to testing. The lead clinician investigator (DM) served as the ordering clinician for the testing at all three centers. A limited number of providers using the imaging centers opted out of having their patients participate. Only patients (including those with a previous history of breast cancer) undergoing routine breast imaging, either by mammogram or ultrasound, were included. Patients undergoing imaging for newly diagnosed breast cancer were excluded.

Clinical, demographic, and family cancer history information were ascertained through test requisition forms and family cancer history questionnaires completed by the patients. Patient questionnaires included clinical questions needed for Tyrer–Cuzick breast cancer risk assessment. Race/ethnicity (ancestry) was self-reported. National Comprehensive Cancer Network (NCCN) guidelines for Genetic/Familial High-Risk Assessment: Breast, Ovarian, and Pancreatic Cancer [24] and Lynch Syndrome (LS) were reviewed to determine if genetic testing would have been guideline indicated for a particular patient [25]. For the patients unaffected with breast cancer who completed the clinical portion of their questionnaire for breast cancer risk assessment, a Tyrer–Cuzick breast cancer risk 5 years and lifetime risk score was calculated [26]. This score was only reported back to patients if their genetic testing (described below) was negative/uninformative for PVs associated with increased breast cancer risk. It was not available if enough information about personal/family history was not provided to compute a Tyrer–Cuzick score or the patient had a personal history of breast cancer.

If the patients’ clinical and/or family history met NCCN guidelines for hereditary cancer testing, they were given the option to request insurance coverage for testing or self-pay. Patients who did not meet guidelines were offered the option to self-pay and financial assistance options were available to eligible patients, based on their income and family size. Genetic information sessions performed by board-certified genetic counselors (Natera, Inc.) were available to patients on a pre- and post- test basis. All patients with a PV in a breast cancer-associated predisposition gene were offered in person risk counseling by the lead clinician investigator (DM).

This study was granted a waiver of consent process under 45 CFR 46.116(d), a waiver of the requirement for documentation of informed consent according to 45 CFR 46.117(c)(2), and a waiver from the HIPAA Authorization Requirement according to 45 CFR 46.164.512(i) (Salus IRB, ID# 21204—01A).

### Hereditary cancer testing

Next-generation sequencing (NGS)-based hereditary cancer risk assessment was carried out utilizing a multiplex gene panel (40 or 53 genes) testing (Empower™, Natera, Inc. in collaboration with Baylor Genetics). The targeted regions of the genes associated with hereditary cancer syndromes are enriched using a capture-based method and sequenced by next-generation sequencing (NGS) using the Illumina platform. The variants detected in exons and within 20 bp of the exon/intron boundary are reported, unless otherwise specified. Read depth analysis is used to detect copy number variation (CNV) for genes. Positive sequencing results from certain genes or regions with highly homologous sequences in the genome are confirmed by gene-specific long-range PCR and Sanger sequencing. Multiplex ligation-dependent probe amplification (MLPA), PCR-based methods, and/or array comparative genomic hybridization (aCGH) are used to confirm copy number changes involving the genes in the test.

All patients underwent testing for at least 25 clinically actionable genes (Table 1). Genes were considered clinically actionable based on the presence of established NCCN and/or peer-reviewed consensus management recommendations for enhanced surveillance, or risk-reducing interventions, and family cascade testing if a PV was detected. Clinically actionable genes were categorized as “high risk” or “moderate risk” based on their reported relative risk for cancer, relative risk of > 4, and relative risk of 2–4, respectively (Table 1). Testing included both HBOC and Lynch syndrome (LS) genes (NCCN HBOC, NCCN Colorectal Cancer (CRC)). Variants were classified consistent with guidelines from the American College of Medical Genetics and Genomics and the Association for Molecular Pathology as previously described [26–28]. Only likely pathogenic and pathogenic variants (PVs) were considered in the analysis: benign and likely benign variants, and variants of unknown significance were not considered.

**Table 1:** List of 25 actionable genes reported for all patients

High-risk Genes	Moderate-risk Genes
*APC*	*ATM*
*BRCA1*	*BARD1*
*BRCA2*	*BRIP1*
*BMPR1A*	*CHEK2*
*CDH1*	*NF1*
*EPCAM*	*RAD51C*
*MEN1*	*RAD51D*
*MLH1*	
*MSH2*	
*MSH6*	
*MUTYH (Biallelic)*	
*PALB2*	
*PMS2*	
*PTEN*	
*SMAD4*	
*STK11*	
*TP53*	
*VHL*	

For the purposes of the current analysis, variants were not considered clinically actionable (i.e., had no potential impact on patient care) and were not included if they:

1. Were in genes only associated with autosomal recessive disease association (e.g., monoallelic *MUTYH* carriers which may conflate estimates of pathogenic variant prevalence) or
2. Were low penetrance variants in clinically actionable genes, such as *CHEK2* c.470 T > C [p.Ile157Thr].

### Analysis

The sociodemographic characteristics and personal and family history of cancer of the study population were explored. Patients’ characteristics were stratified based on the presence and type of PV. The prevalence of P/LP variants was calculated. For patients with a P/LP variant, the proportion who did and did not meet NCCN guidelines for genetic testing was evaluated. We also evaluated the proportion of patients with PV who would have qualified for additional screening based on the empiric risk model (i.e., Tyrer–Cuzick score > = 20%).

## Results

### Study population

A total of 1,943 women undergoing breast imaging elected to have hereditary cancer genetic testing during the study period (Table 2). Median age was 66 years (range 18–89 years). Self-reported race and ethnicity were Asian 5.0% (*N* = 85); Black 20% (*N* = 339); White 38% (*N* = 650); and Hispanic 32% (*N* = 534).

**Table 2:** Characteristics of individuals who underwent genetic testing (*N* = 1943)

Characteristics	Patients with a PV in HBOC gene N= 33	Patients with a PV in a LS gene N= 6	Patients without a PV N= 1904	All N= 1943
Median age in years (Range)	61 (36–78)	67 (19–73)	65 (18–89)	66 (18–89
< 50 years, n (%)	5 (15.2)	2 (33.3)	349 (18.3)	356 (18.3)
≥ 50 years, n (%)	28 (84.8)	4 (66.7)	1555 (81.7)	1587 (81.7)
Race and Ethnicity, n (%)				
Black	2 (6.1)	1 (16.7)	336 (20)	339 (20)
White	14 (42.4)	2 (33.3)	634 (38.0)	650 (38)
Asian	3 (9.1)	0 (0.0)	82 (5.0)	85 (5.0)
Hispanic	8 (24.2)	1 (16.7)	525 (32)	534 (32)
Multiple races selected	1 (3.0)	0 (0.0)	23 (1.4)	24 (1.4)
Other	1 (3.0)	1 (16.7)	56 (3.4)	58 (3.4)
Missing	4 (12.1)	1 (16.7)	248 (13.0)	253 (13.0)
Personal history of cancer (any type)	1 (3.0)	0 (0.0)	145 (7.6)	146 (7.5)
Family history of cancer (in patients with no personal cancer history)	25 (75.8)	6 (100.0)	791 (41.5)	822 (42.3)
No known personal or family history of cancer	7 (21.2)	0 (0.0)	968 (50.8)	975 (50.2)

HBOC, Hereditary Breast and Ovarian Cancer-related genes *(BRCA1, BRCA2, PALB2, ATM, CHEK2, BARD1, NF1, BRIP1)*

LS, Lynch syndrome genes *(MSH2, PMS2)*

**Fig. 1:**
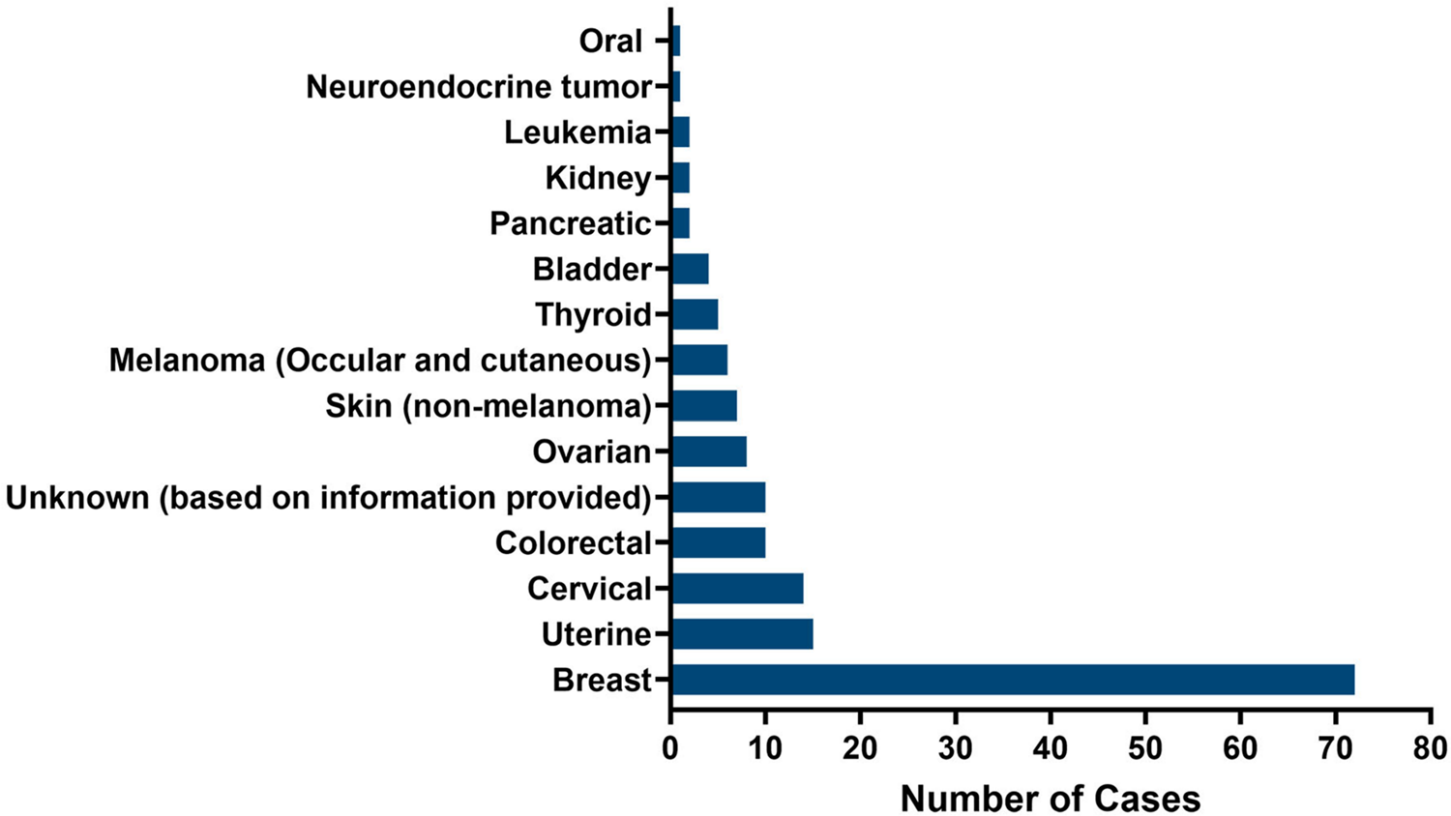
Personal cancer history in the full cohort (*N* = 1943)

A personal history of cancer was documented for 7.5% (*N* = 146) (Fig. 1), a family history of cancer in 42.3% (*N* = 822), and no personal or family history of cancer in 50.2% (*N* = 975). A personal history of breast or ovarian-related cancers was recorded in 4% (*N* = 80), endometrial cancer in 0.8% (*N* = 15), and colorectal cancer in 0.5% (*N* = 10).

Overall, 18.2% (354/1943) of patients met current NCCN guidelines for hereditary breast and ovarian cancer (HBOC) gene testing, 3.7% (71/1943) met NCCN guidelines for Lynch syndrome genetic testing, and 1.0% (19/1943) met both HBOC and Lynch syndrome guidelines for testing (Table 3).

**Table 3:** Number (percentage) of patients who met NCCN criteria for hereditary cancer testing based on personal and/or family history

Characteristics	Patients with a PV in HBOC gene N= 33 (%)	Patients with a PV in a LS gene N= 6 (%)	Patients without a PV N= 1904 (%)	All N= 1943 (%)
HBOC	10 (30.3)	3 (50.0)	341 (17.9)	354 (18.2)
Lynch	1 (3.0)	0 (0.0)	70 (3.7)	71 (3.7)
Both HBOC and Lynch	1 (3.0)	0 (0.0)	18 (0.9)	19 (1.0)
Pre-test Tyrer-Cuzick* risk score calculated, n (%)	25 (75.8)	4(66.6)	1405 (73.8)	1434 (73.8)
Pre-test Tyrer-Cuzick* risk >20%	2 (8.0) *	1 (16.7)	66 (6.0)	67 (4.7)

*Pre-test Tyrer–Cuzick score was calculated for the patients with no prior history of breast cancer

### Prevalence of pathogenic variants

**Fig. 2:**
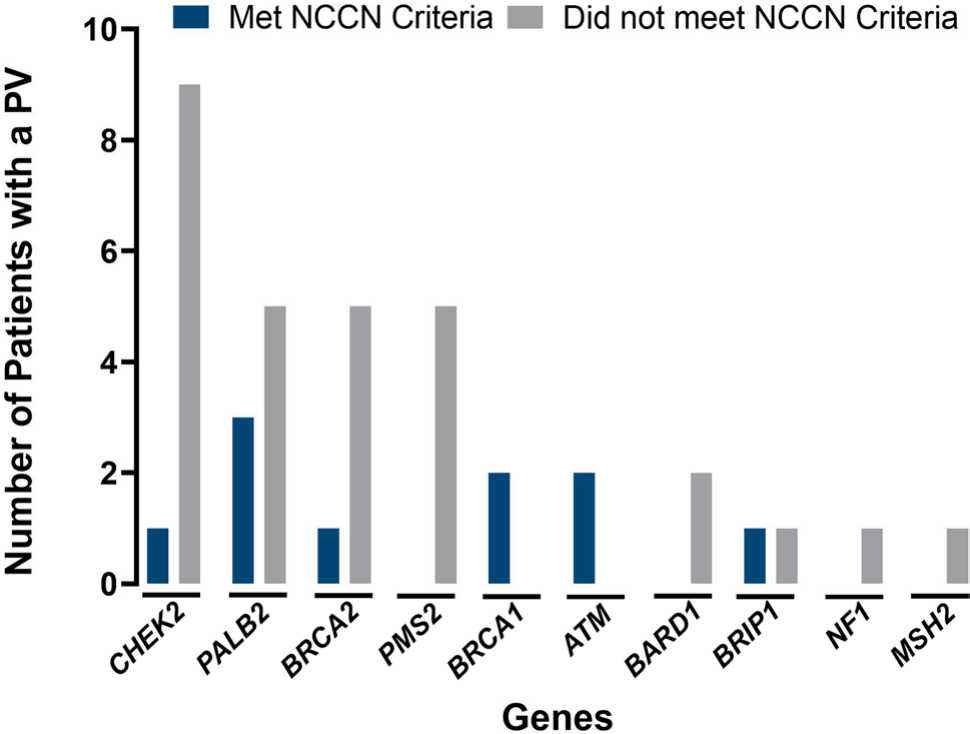
Number of clinically actionable PVs identified in high- or moderate-risk genes in patients who either met or did not meet NCCN guidelines for testing of the indicated gene

Among 1943 patients who received genetic testing, 39 (2%) were identified as carriers of a PV in an autosomal dominant clinically actionable HBOC-related or LS gene (Table 3). Of the 39 PVs identified, 84.6% (*N* = 33) were in HBOC-related genes which corresponds to a prevalence of 1.7% (33/1943) in the total cohort. The remaining 15.4% (*N* = 6) PVs were in LS genes which corresponds to a prevalence of 0.3% (6/1943) in the total cohort. The most common PVs were in *CHEK2* (10/39; 25.6%); *PALB2* (8/39; 20.5%); *BRCA2* (6/39; 15.4%); and *PMS2* (5/39; 12.8%) (Fig. 2). Of the 34 PVs where race/ethnicity were known, 47% were detected among non-White patients (Table 2). The PV prevalence (%) was distributed across the respective ancestral groups as follows: Black 3/339 (0.89%); White 16/650 (2.4%); Asian 3/85 (3.5%), and Hispanic 9/534 (1.7%).

Patients with a PV were over 50 years of age at the time of their testing in 82.1% (32/39) of cases. This is similar to the 1587/1943 (81.7%) of patients aged 51 or older who were tested in the total cohort and consistent with National screening guidelines for women at average risk [27] (Table 2).

### Guideline eligibility for genetic testing or enhanced surveillance breast cancer

Only 38.5% (15/39) of PV carriers met either NCCN guidelines for HBOC testing or LS testing prior to genetic testing. Of these, 25.6% (10/39) met HBOC criteria for genetic testing with PVs in HBOC-related genes and 7.7% (3/39) had a PV in an LS gene, and 5.1% (2/39) met criteria for LS testing with PV in HBOC-related genes.

Notably, 5 out of 6 patients with a *BRCA2* PV and 5 out of 8 patients with a *PALB2* PV did not meet criteria for HBOC testing. The frequencies of clinically actionable PVs identified in high- or moderate-risk genes in patients who either met or did not meet NCCN guidelines for inclusion of the genes are shown in Fig. 2.

Data allowing calculation of the patient’s Tyrer–Cuzick score were available for 64.1% (25/39) of patients with a PV in an autosomal dominant clinically actionable HBOC (Table 3). Of these, only 8% (2/25) had a Tyrer–Cuzick score ≥ 20 which would have triggered health insurance coverage for increased surveillance for breast cancer, absent in the PV finding (Table 3).

## Discussion

We found that 2% (39/1943) of women undergoing hereditary cancer gene testing as part of their clinical care at the time of breast imaging had PVs in hereditary cancer genes that had implications for cancer surveillance and clinical management. Importantly, the population undergoing testing was racially and ethnically diverse compared to previous studies [20, 28], predominantly over the age of 50 and without a personal history of cancer (92.5%). This was similar or more diverse compared to CDC population data on race and ethnicity for females aged 55 to 74 years who were resident in Texas in 2021 (4.8% Asian, 12.5% Black, 80.7% White, and 28.1% Hispanic) [29].

Among women who had a PV in either a HBOC or LS gene, only 38.5% met NCCN guidelines for testing of either of these conditions. Of those who had a PV in an HBOC gene and had data needed for a Tyrer–Cuzick score, only 8% had an estimated lifetime score of ≥ 20%. Therefore, while testing as part of clinical care at the time of breast imaging identified some women who were eligible for hereditary cancer gene testing based on NCCN guidelines, it predominantly identified women with PVs who neither met NCCN guidelines for testing nor the Tyrer–Cuzick threshold for increased surveillance for breast cancer [30]. Consequently, without this opportunity for genetic testing, women with PVs may not have accessed risk-appropriate enhanced surveillance.

Identification of individuals with PVs in HBOC and LS genes provides opportunities for cancer prevention and earlier detection [13, 31]. For people with PVs in HBOC genes, there are recommendations and options for earlier mammography, screening breast MRI, chemoprophylaxis, and risk-reducing surgeries, such as mastectomy and bilateral salpingo-oophorectomy [8, 10, 11, 13]. Knowledge of specific PVs can also impact decisions about surgery and adjuvant therapies [32–34]. The median age of the population in the current cohort was 66 years and previous research has suggested that HBOC gene testing in younger women would be the most cost-effective approach to testing [35]. Nonetheless, a study of the remaining lifetime risk for the subset of women who were older than 65 years in the population-based CARRIERS project [36] indicated that *BRCA1*, *BRCA2*, and *PALB2* PVs were associated with enough breast cancer risk to warrant high-risk screening [37]. For people who have PVs in LS genes, there is also compelling evidence of the benefit of colonoscopy in reducing mortality from CRC [9, 38]. Additionally, in women with PVs in LS genes, the lifetime risk of endometrial cancer is similar to that of CRC, which can largely be prevented by hysterectomy [39]. For women with PVs who chose to share this information with family members, it can provide more accurate risk assessment and cascade testing [14–16]. The yield of PVs is typically higher (~ 10%) [13] when we use the guideline criteria to screen the eligibility for genetic testing. However, this study demonstrated a meaningful yield (~ 2%) of clinically actionable PVs among participants who did not meet any guideline. Amplifying this point, 5 out of 6 *BRCA2* carriers identified by this unselected approach did not meet any guideline and there is ample evidence of reduction in cancer-specific and all-cause mortality by standard of care gene-specific clinical management [8, 40, 41]. Thus, the universal testing approach and increasingly cost-effective genetic testing are likely to have a high impact despite a modest yield.

Perhaps one of the most striking findings in this clinical cohort was the diversity of the population that accessed the testing. Later stages of cancer at the time of diagnosis and lower cancer survival rates are clearly documented in Black people in the USA [42]. There are data indicating that people of color or Hispanic ancestry are less likely to be referred for genetic counseling or testing and are less likely to take up genetic testing in the absence of a personal history of cancer [20, 42, 43]. As demonstrated in our study, increasing access and convenience of testing may help overcome barriers to more equitable testing [43, 44].

Limitations of this study include that women under 40 years of age without documented increased risk are unlikely to have routine mammography and therefore, may not be represented in this cohort. Thus, *BRCA* carriers may be under-represented. Nonetheless, several *BRCA2* carriers, the majority of which did not meet any testing guidelines, were identified representing a critical opportunity for screening and prevention. Another limitation of the current cohort is that the number of patients who declined hereditary cancer testing or who may have had genetic testing prior to this study was unknown. Given the uncertainty about the total number of women who received the invitation to have genetic testing, the potential benefit of our strategy with regard to the yield of actionable PVs in the imaging center population could be over- or underestimated. Finally, though the racial and ethnic composition of the study participants were exceptionally diverse, we do not know if there were significant differences in the uptake among the respective groups [45]. Nonetheless, (as cited above) the population who underwent testing is diverse and generally representative of the ethnic makeup of the state of Texas. Further, we do not have qualitative or quantitative data on how the patients made their decision to participate and receive testing as it was beyond the scope of our study. However, we believe that this will be important for future research in the context of population health implementation. Finally, the presence of a PV or elevated empiric risk (e.g., > 20%) does not guarantee insurance coverage, and access has been problematic across different healthcare systems and among the underinsured. Nonetheless, we believe that this report provides additional evidence supporting access to risk-appropriate care. Without granular insurance data, we note that all of the individuals in the study at the least had access to the imaging centers.

Additionally, while genetic information sessions both before and after testing were available to all patients who underwent testing, formal pre-test genetic counseling was not required. Requiring pre-test genetic counseling may itself present a barrier to testing [46], so there may be a trade-off between the benchmark of full-genetic counseling and the use of abbreviated genetic information sessions to improve access to potentially life-saving information, especially in population health settings. Additional research is needed to establish what constitutes optimal pre- and post-test counseling and informed consent for patients receiving genetic testing in non-genetic/population health settings. However, in the meantime, the results of this and similar studies suggest that testing in a diverse imaging center population can extend the reach of genetic cancer risk assessment, has a clinically meaningful and actionable yield of cancer-associated PVs, and can help address inequity in access to testing and risk-appropriate screening and prevention.

## Data Availability

This is descriptive summary data on genetic testing outcome. The specific variant level data are contributed to ClinVar by Baylor Genetics, Houston, Texas.
